# Evolution of rough-surface geometry and crystalline structures of aligned TiO_2_ nanotubes for photoelectrochemical water splitting

**DOI:** 10.1038/s41598-018-29247-3

**Published:** 2018-07-18

**Authors:** Maryam Zare, Shahram Solaymani, Azizollah Shafiekhani, Slawomir Kulesza, Ştefan Ţălu, Miroslaw Bramowicz

**Affiliations:** 1 School of Physics, Institute for Research in Fundamental Sciences (IPM), P.O. Box 19395-5531 Tehran, Iran; 2 Young Researchers and Elite Club, West Tehran Branch, Islamic Azad University, Tehran, Iran; 3 Physics Department, Alzahra University, Tehran, 1993891167 Iran; 4 University of Warmia and Mazury in Olsztyn, Faculty of Mathematics and Computer Science, Sloneczna 54, 10-710 Olsztyn, Poland; 5 Technical University of Cluj-Napoca, The Directorate of Research, Development and Innovation Management (DMCDI), Constantin Daicoviciu St., no. 15, Cluj-Napoca, 400020 Cluj county Romania; 6 University of Warmia and Mazury in Olsztyn, Faculty of Technical Sciences, Oczapowskiego 11, 10-719 Olsztyn, Poland

## Abstract

Nowadays, increasing awareness of environment and fossil fuels protection stimulates intensive research on clean and renewable sources of energy. Production of hydrogen from water through solar-driven splitting reactions is one of the most promising approaches in the field of photoelectrochemistry (PEC). In this work we have fabricated well-aligned, highly-ordered, smooth-mouth TiO_2_ nanotube arrays (TNAs) in a two-step anodization process of titanium foil, which were then used as photoelectrodes for PEC water splitting. It demonstrates for the first time correspondence between non-linear component characteristics of multiscale rough surface and crystalline structure of annealed TNAs measured at various fabrication stages and their photoelectrochemical response. The as-anodized TNAs with isotropic surface (deduced from AFM and SEM images) and largest figure of merit (according to their PEC performance) were annealed at 450 °C in air. Scale-invariant descriptors of the surface structure of the deposits involved: fractal dimension, corner frequency, roughness, size of nanostructures and their dominant habits. Moreover, X-ray diffraction data processed using the Rietveld method confirmed co-existence of various oxides, for example: TiO_2_ in the form of anatase, TiO and Ti_3_O_5_ phases in the TNAs under study pointing that previous well-established mechanisms of the TNA growth were to certain degree incomplete.

## Introduction

Photoelectrochemical (PEC) water splitting is one of the most favorable approaches for H_2_ production as a clean energy vector of the future. Since the work by Fujishima and Honda in 1972^1^, increasing research has been carried out towards this issue using electrodes made of various materials, e.g. semiconductors. Unfortunately, their practical application has encountered a number of technical complications. Among the metal oxides that has been taken into consideration, titanium dioxide (TiO_2_) is found to be promising in PEC water splitting^2–9^ due to its appropriate band-gap structure, superior chemical and optical stability and low cost. In particular, TiO_2_ nanotube arrays prepared in anodization processes have numerous advantages over TiO_2_ nanoparticle films resulting from facile preparation procedure, high surface-to-volume ratio for contact with the electrolyte, large light harvesting efficiency improved by light scattering into tubular morphology, and high electron mobility induced by their unidirectional channel^10,11^. Some strategies such as doping or semiconductor heterocoupling were used for modification of TiO_2_ nanotube arrays to be activated under visible light^12–14^.

In a conventional one-step anodization process, prolonged etching of the tops of the tubes usually leaves inhomogeneous and rough surface of the specimen. The debris on the mouths of nanotubes were found to form nanograss or nanoneedles structures leading to underprivileged alignment within nanotube arrays^11^. The reason for this effect is that the walls of the tubes become thinned at their top ends, so that they cannot support their own weight or withstand capillary forces when drying. Consequently, they collapse and bundle in the form of nanograss which could close the mouths of nanotubes and in turn decrease the effective surface area of TNA, avert charge carrier direction to the substrate and passivate active sites for chemical reactions. To overcome this problem, several effective approaches towards production of highly ordered and uniform TiO_2_ nanostructures have been developed^15–19^. Among them, the two-step anodization of Ti foils turned out to be the most convenient and feasible method^19–21^. In this process, TNAs are deposited and subsequently removed after first anodization stage, and during the second stage new TNAs are grown over the same dimpled substrate. Obviously, the dimples play the role of nuclei for the growth of nanotubes that neither collapse nor form bundles. Recent report has shown that defective TiO_2-x_ layer is formed between Ti substrate and TiO_2_ nanotube arrays in this fabrication procedure introducing a new vacancy band below conduction band expanding the photon-absorbance of TiO_2_ into the visible light region^22^. Nevertheless, convincing evidence on the spatial microstructure morphologies and fractal characteristics of each grown layer and defective sublayers at the interface of substrate/TiO_2_ nanotube arrays (TNAs) junction are still lacking.

To date there are hardly known experimental works related to the issue of proper characterization of surface irregularities of TNAs defined by its large surface-to-volume ratio, which are extremely important in determining various surface phenomena influencing efficiency of the photoelectrolysis. Surface roughness and geometry appear key factors in that aspect concerning published results for TiO_2_ in various nanostructural forms (powders, tubes, aligned tubes etc.) Unfortunately, statistical parameters used to characterize surface asperities are scale-dependent and cannot be determined uniquely. To deal with multiscale property, non-linear methods need to be used, such as fractal analysis showing the importance of scale-invariant components of the surface roughness: roughness exponent, lateral correlation length, long-distance roughness and high-order regularities. Previous studies demonstrated reliability of the fractal analysis in determination of important spatial features of nanostructured materials^23,24^. In addition, fractal analysis proved to be useful in studies of morphology and mechanical properties of various structures upon heat treatment^25,26^, nanomechanical processing^27,28^, in contact with body tissues and fluids etc^29,30^.

In the current paper, we present an improved two-step anodization process for the fabrication of well-aligned, highly-ordered and smooth-mouth TiO_2_ nanotube arrays on Ti substrate with detailed investigations of morphological and crystalline structures of synthesized nanostructures at each fabrication steps. Then, we utilized and comprised obtained structures as electrodes for PEC water splitting to explore the flat-band potential and energy band alignment. To verify how different structural and morphological properties of TNAs affect electronic structure, photoelectrochemical performance of TNA layers after various fabrication stages are tested for water splitting reaction. Correlations between PEC performance and scalable, component roughness at the nanolevel have been studied systematically for the first time.

## Experimental Details

### Synthesis of TNAs

**Figure 1:**
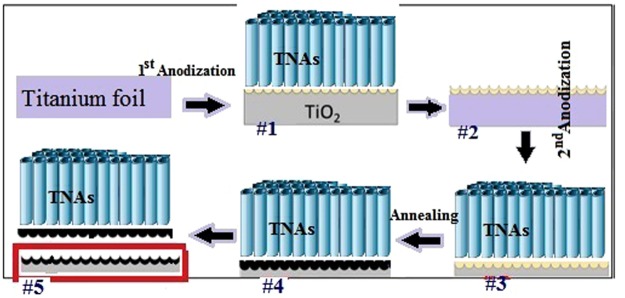
Experimental procedure for TNA fabrication.

Highly ordered TiO_2_ nanotube arrays were fabricated by electrochemical anodization of titanium foil (>99% purity, 0.025 mm thickness). The foil was cut into small rectangles 3 cm long and 1 cm wide, and these samples were cleaned by sonication in, subsequently: acetone, ethanol and DI water, each of which took 10 minutes. Pretreatment procedure was completed with drying in hot air stream. Anodization was carried out in a conventional two-electrode setup on clean Ti substrates as working electrodes and another rectangular Ti sheet (>99% purity, 3 × 1 cm^2^ area, 0.8 mm thickness) as the counter-electrode. Solution of 0.3 wt. % NH_4_F (Sigma-Aldrich) and 2 vol. % H_2_O in ethylene glycol (Merck) was used as an electrolyte. The structures were fabricated in a multi-step procedure and once a given step was completed, the samples were picked out for surface characterization. Firstly, long anodization was performed that took 1 h at 50 V supplied by a potentiostat (Sample #1). Then, the sample was rinsed in a 1:1 (vol.) mixture of ethanol and deionized (DI) water and sonicated for 5 min with 400 W ultrasounds power (Sample #2). This treatment removed as-grown nanotubes and ended up with the dimples on the substrate surface. Subsequently, dimpled substrates were subject to second anodization in the same electrolyte, at the same voltage (50 V), but for 30 minutes only (Sample #3). Both fabrication steps were carried out in room temperature and room light. After that, the samples were cleaned with ethanol and DI water and left for drying in air. Then, the as-anodized TNAs were annealed in air at 450 °C for 3 h with a heating rate of 2 °C/min (Sample #4). Finally, the annealed TNA layers were peeled off by a blade in order to reveal the blue defective underneath layer (Sample #5). Schematic picture of the fabrication procedure is shown in Fig. 1. Details and ID of samples are listed in Table 1.

**Table 1:** Description of experimental procedure for TNA fabrication.

Sample ID	Description
#1	1^st^ stage TNA (after 1 h anodization)
#2	Dimpled sublayer
#3	2^nd^ stage TNA (after 30 min anodization)
#4 (TNA electrode)	Annealed TNA
#5	Dimpled sublayer (after annealing)

### Characterization methods

The surface and cross-sectional morphologies of TiO_2_ nanotube arrays were characterized by means of the Field-Emission Scanning Electron Microscopy and transmission Electron Microscopy (FESEM, Hitachi S-4160 and TEM, Philips CM30). The crystalline structure of the deposits was investigated using X-ray diffraction with CuK_α_ radiation (0.15406 nm) for 2θ angles from 30 to 90° (STOE-XRD). Autoprobe M4 atomic force microscope (AFM, Veeco) equipped with ProScan V1.51b software (Veeco) was employed to study the surface of samples.

All photoelectrochemical measurements (OCP and LSV) were performed using Origalys potentiostat (Origalys, France) connected with a cell filled with 500 ml of 0.5 M KOH as electrolyte solution. The cell was equipped with three electrodes: working electrode (WE) made of deposited TNAs and re-shaped into square with 1 cm side length, Ag/AgCl as a reference electrode (RE), and platinum rod as a counter-electrode (CE), respectively. Dark experiments were performed at room light, while the illumination with 400 mW/cm^2^ intensity was applied by a Xe light source (EIKI, Japan) through quartz window through WE in illuminated experiments. The linear sweep voltammetry measurements (LSV) were carried out with the scan rate of 0.1 V/s in the potential range from (−1) to 2.5 V vs. Ag/AgCl reference.

Spatial characteristics of investigated TNAs can be derived independently from AFM and SEM images making use of the allometric similarity laws. Although AFM images provide direct information on surface topography, whereas SEM provide pseudo-height gray-scale data, both datasets can be analyzed in the same manner. Numerical procedure begins with calculation of the autocorrelation function R according to the formula^31^:

1 $${R}_{mn}=\frac{1}{(N-n)(N-m)}\sum _{p=1}^{N-n}\sum _{q=1}^{N-m}({z}_{p+n,q+m}\cdot {z}_{p,q})$$

where (m, n) enumerates discrete shift between the image and its lagged copy. Autocorrelation becomes zero for infinite lags, and the ratio of extreme decay lengths τ defines the surface anisotropy ratio S_tr_^32^:

2 $$0 < {S}_{tr}=\frac{{\tau }_{a1}}{{\tau }_{a2}}\le 1$$

where: a_1_, and a_2_ – are the directions of the fastest and the slowest autocorrelation decay, respectively.

In the next step, autocorrelation function is converted into the structure function according to^33^:

3 $${S}_{mn}=2{S}_{q}^{2}(1-{R}_{mn})$$

where S_q_ is the surface roughness. Mean profile of the structure function averaged around origin exhibits specific scaling behavior^34^:

4 $$S(\tau )=K\cdot {\tau }^{2(2-D)}$$

where D – is the fractal dimension, and K – pseudo-topothesy. In general, D and K correspond to the way, how the relative and absolute amplitudes of surface height variations depend on the wavelengths, respectively. On the other hand, the corner frequencies τ_cf_ are thresholds, at which the scaling law changes qualitatively.

Physical properties of the materials under investigation can be also studied making an insight into the arrangement of the crystal lattice. An effective way towards atomic structure of the materials is via X-ray diffraction techniques. However, the drawback of this method in terms of materials with apparent mosaic structure is that the diffraction peaks overlap and the proper crystal structure cannot be easily determined using Braggs’ law solely. In order to reconstruct accurate crystal lattice and determine the contributions of chemical compounds that form given structure, the Rietveld method^35^ was used in this work. Apart from that, the diameters of the coherently-diffracting domains (CDD) responsible for scattering of X-ray beams and the relative deformations of crystal lattices are determined from the Williamson-Hall plot.

## Results and Discussion

### AFM and SEM Characterization

**Figure 2:**
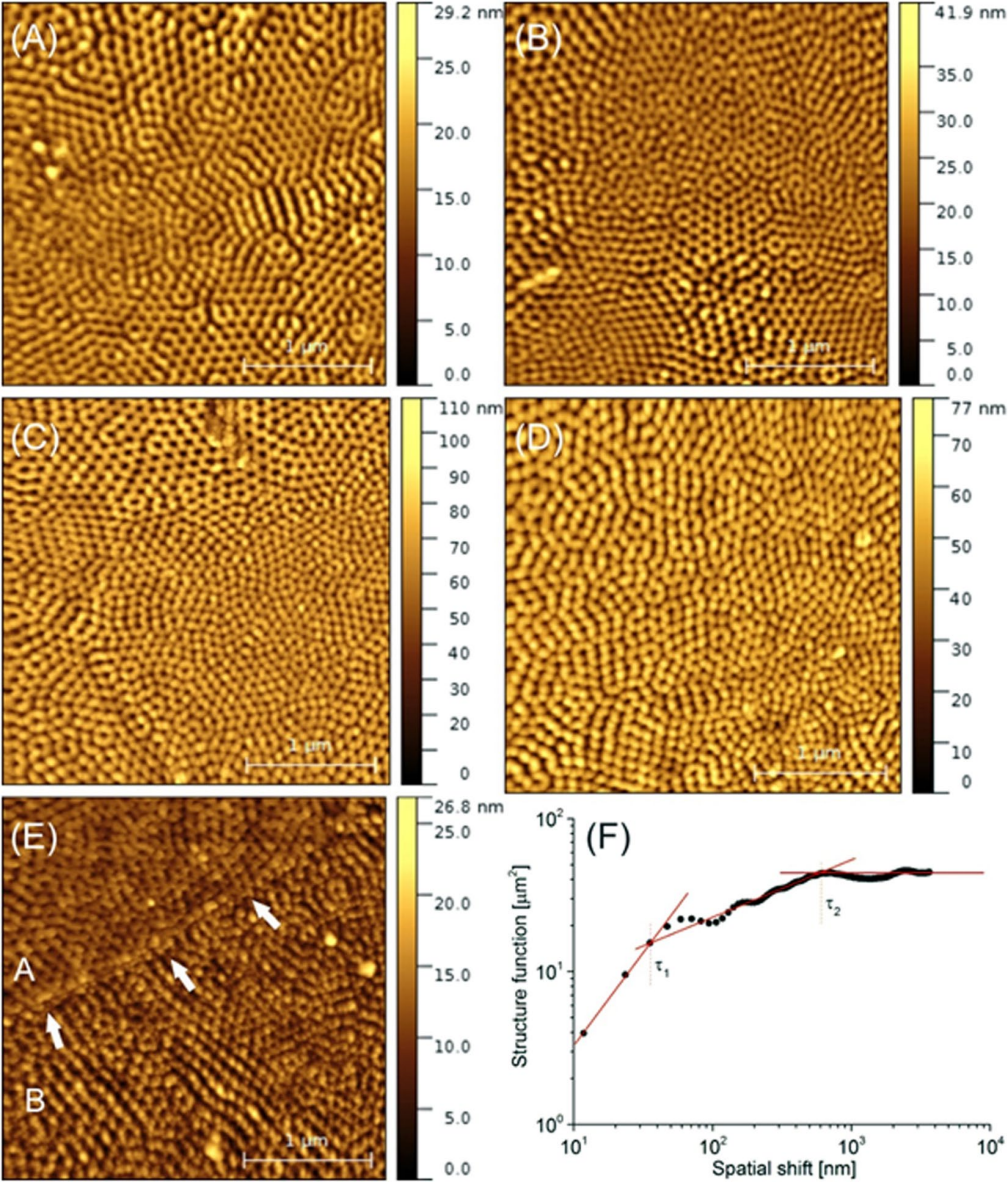
AFM images of TNAs at various stages of their fabrication process taken with 3 µm scan length: (**A**) 1^st^ anodization, (**B**) dimpled substrate without as-grown nanotubes, (**C**) 2^nd^ anodization, (**D**) annealing, (**E**) dimpled film without nanotubes, (**F**) plot of the structure function (bifractal characteristics) of the TNAs at the final stage. Arrows in (**E**) mark the boundary separating the structures with dominant rings (**A**) and chains (**B**).

AFM images shown in Fig. 2A–E exhibit non-homogeneously distributed structures built from tiny grains 40–50 nm in diameter. These structures typically take shape of hexagonal rings or linear chains, hence the TNAs possess numerous dislocation and point defects as well as vacancies. The thickness of the chains equals the diameter of the grains, whereas the nearest-neighbor chains are as far as twice of that, i.e. 90–100 nm, which also applies for the diameter of the rings.

**Figure 3:**
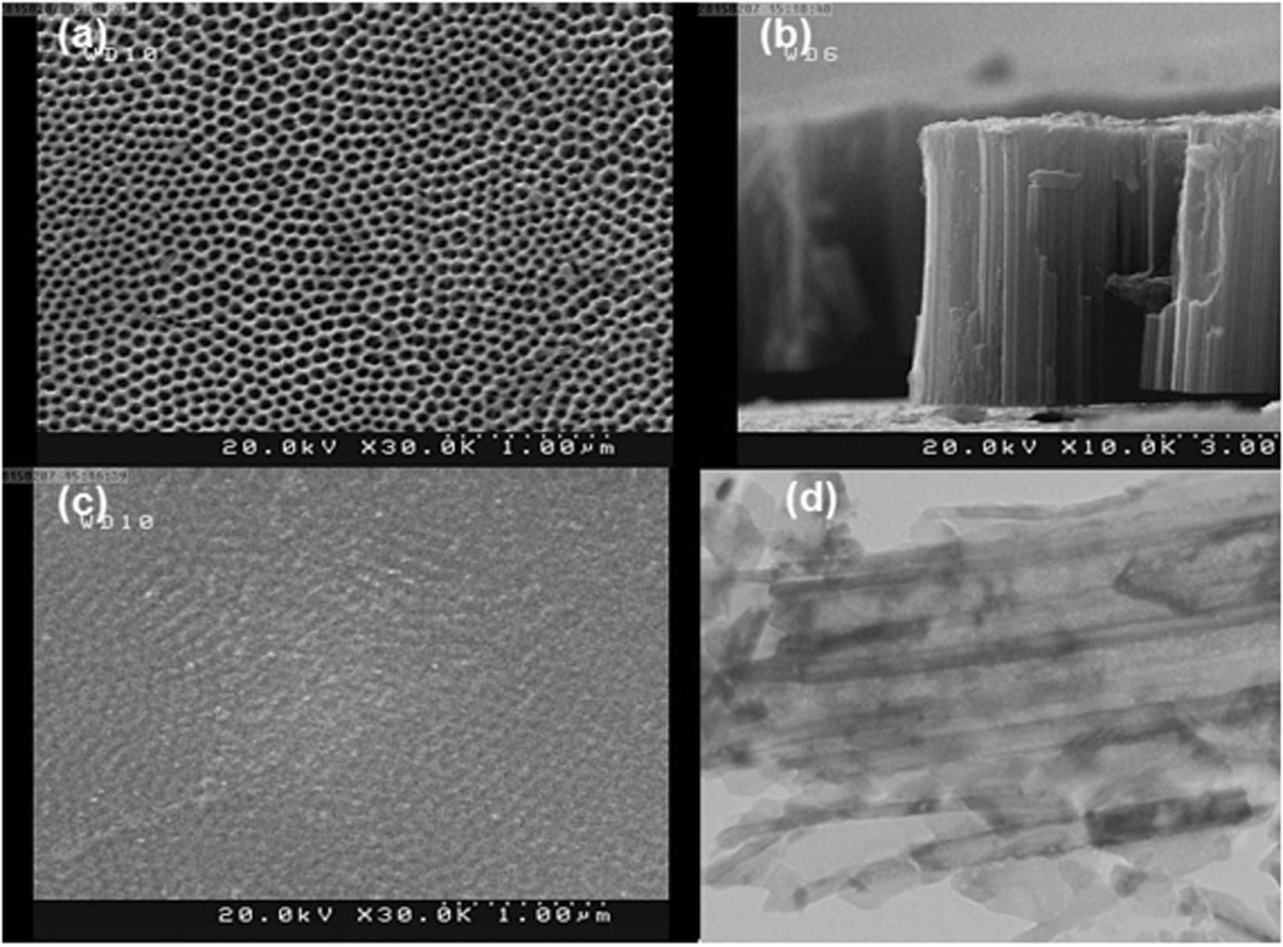
SEM images of TNAs at two latest stages of their fabrication process: (**a**) annealing, (**b**) annealing (cross-section), (**c**) dimpled film without nanotubes. (**d**) TEM image of removed nanotubes.

Table 2 summarizes surface texture parameters obtained for TNAs structures derived from AFM images. All the structures under study seen in Fig. 2 except for the sample #5 exhibits monofractal characteristics considering their self-similar behavior, however, this scaling law is limited to very short wavelengths established by the corner frequency (42–47 nm), comparable to those of the grain diameter (40–50 nm). Such a result is a sign of a short-range spatial alignment, which together with a very large anisotropy ratio between 0.90 and 0.95, strongly suggest that the grains form chain/ring aggregates in a random manner. In addition, this sample exhibits moderate fractal dimension that varies from 2.37 to 2.45 indicating moderate development of its residual surface. In contrast, sample #5 exhibits bifractal characteristics, which is a clear indication of a second-order spatial alignment. Figure 3f presents specific log-log plot of the structure function with three different slopes separated by two corner frequencies. Note that the lower fractal dimension that corresponds to the scaling behavior of single grains is similar to those in previous samples, and the lower corner frequency is only slightly smaller than previously (35 vs. 45 nm). On the other hand, the upper fractal dimension corresponding to the self-similar scaling of the entire aggregates approaches 2.81, which means that relative amplitude of the surface height variations is a steeper function of the wavelength. Such a behavior can be observed as long as the spatial shift does not exceed 600 nm, that is an order of magnitude larger than the grain diameter. Very low anisotropy ratio equal to 0.26 specific of highly anisotropic forms agrees well with observation of the subtle, long-range aggregate structure of the elemental ring/chain units. This is probably due to spatial separation of both compounds seen in Fig. 2E. White arrows in Fig. 2E mark the boundary between layers with different dominant structural forms: hexagonal rings (A) and linear chains (B).

**Table 2:** Fractal and statistical descriptors of the TNAs structures derived from AFM images: D – fractal dimension, τ – corner frequency, S_tr_ – anisotropy ratio, S_q_ – surface roughness.

Sample	D_1_	D_2_	τ_1_ [nm]	τ_2_ [nm]	S_tr_	S_q_ [nm]
#1	2.37	—	48	—	0.94	4.0
#2	2.38	—	42	—	0.94	5.5
#3	2.44	—	45	—	0.95	15
#4	2.43	—	46	—	0.90	11
#5	2.41	2.81	35	600	0.26	4.7

Results obtained using SEM imaging are similar to those from AFM. Figure 3 presents SEM images of the selected samples under study, i.e. taken after annealing procedure (Fig. 3a,b) and after the removal of the upper layers (Fig. 3c). Figure 3d shows the TEM image of removed nanotubes after sonication in ethanol. Figure 3a shows the upper surface of the sample #4. Similar to AFM images, observed TNAs walls are 40 nm thick, and form rings 100 nm in diameter. In turn, Fig. 3b shows the cross-section of the TNAs layer. The average length of the tubes is found to be round 5.5 µm, hence they exhibit large aspect ratio that approaches 55.

Fractal parameters derived from SEM images are summarized in Table 3 turn out to be close to those in Table 2. Let us note that the annealed surface in sample #4 appears monofractal, while uncovered substrate in sample #5 reveals bifractal specification. Both fractal dimension (2.45) and corner frequency (35 nm) in sample #4 are identical to those in AFM image, although the anisotropy ratio is found substantially lower (0.56) due to anisotropy in the ring alignment. On the other hand, both fractal dimensions in sample #5 are larger compared with AFM results, specific of highly developed surfaces of porous media. Lower corner frequency equals to 25 nm, comparable to the grain diameter, while the upper corner frequency that delimits the allometric scaling behavior equals to 1100 nm, and is twice as large as those in AFM images. Large value of the corner frequency is connected with relatively large anisotropy ratio, which approaches 0.49, and doubles with respect to AFM images.

**Table 3:** Fractal and statistical descriptors of the TNAs structures on final stages of their fabrication process derived from SEM images: D – fractal dimension, τ – corner frequency, S_tr_ – anisotropy ratio.

Sample	D_1_	D_2_	τ_1_ [nm]	τ_2_ [nm]	S_tr_
#4	2.45	—	35	—	0.56
#5	2.84	2.97	25	1100	0.49

### XRD Characterization

**Figure 4:**
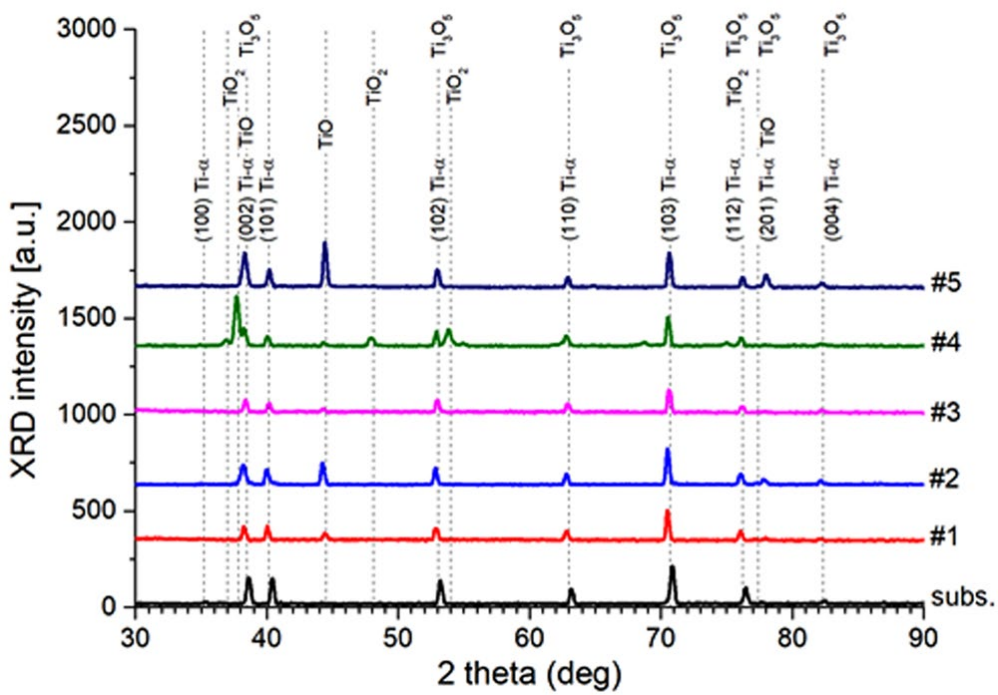
XRD patterns taken from the TNA films under investigation at various stages of their fabrication process. Dotted lines mark respective XRD peaks from stress-free Ti-α crystal lattice (labeled with Miller indices). Similar positions of peaks of other crystalline phases are marked with their names, respectively (“subs.” stands for substrate).

Figure 4 presents XRD patterns taken from the TNAs samples under investigation at various stages of their fabrication procedure. Note the vast number of diffraction peaks labeled in this figure corresponding to a compound structure of the deposit. Apart from pure titanium in the alpha phase it is also possible to observe several oxides: titanium oxide TiO, titanium dioxide TiO_2_ in the form of anatase, and some amounts of Ti_3_O_5_. All the mentioned XRD peaks can be found in 2 theta range from around 30° to 90°, however, several even more distinct XRD lines can be also found in the low-angle range between 10° and 30°. Unfortunately, their origin is not clear, but one might suppose that they reflect higher-order pattern of TNAs arranged in the form of a hyperstructure and they are corresponding to interstitial diffusion or higher-order mosaic domains. This observation is connected with the fact that positions of Ti peaks are generally shifted towards smaller 2 theta angles and hence larger lattice constants. Tensile stresses might be then due to atoms located between lattice sites, i.e. interstitial defects. On the other hand, regular arrangement of observed arrays of TNA (mosaic) might also influence the scattering of X-ray beams. This observation is supported by the fact that the intensity of low-angle peaks sharply increases in samples with uncovered dimples (#2 and #5) with respect to those with strongly anisotropic and possibly highly porous films containing long filaments of nanotubes that suppress incident radiation. These results need to be clarified in future works.

Table 4 summarizes structural data on crystalline phases within TNAs obtained by decomposition of their XRD spectra using the Rietveld algorithm. Phase contribution of polycrystalline titanium in the alpha form sharply decreases during the fabrication process from 100% in the beginning (pristine foil substrate) to 8% after first anodization. Then, it almost vanishes at 1% after cutting-off the overgrown film, but then increases and varies between 5 and 12% during subsequent stages of the process. As a rule, the contribution of the substrate material decreases after each cutting routine, which might be due to the fact that the chemical transformation of native titanium into respective oxides occur in the outer layer of the bulk crystal mostly and that deposited nanotubes still contain some amounts of Ti atoms. The size of the CDD grains monotonically decreases during the process from 250 to 130 Å, and relative lattice deformation remain compressive in the range between 0.11 and 0.33% (Table 4). Anatase is found the main crystalline phase within samples under investigation. Its phase contribution starts from 30% just after first anodization step, and increases even further afterwards. It turns out that the contribution of this oxide is much higher within the uncovered dimples (samples #2 and #5) then within non-annealed nanotubes (samples #1 and #3, respectively). Diffracting domains generally increases their dimensions from 70 to 340 Å throughout the process, although they are strikingly similar in the dimples (90–100 Å). Relative stresses turns from tensile to compressive ones and their absolute value varies between 0.21–0.44%.

**Table 4:** Structural properties of the TNAs structures derived from XRD patterns using Rietveld refinement method and Williamson-Hall plot: a_0_, b_0_, c_0_ – lattice constants, V – relative volume crystalline phase contribution, <D> – diameter of coherently-diffracting domains (CDD), ε – relative deformation of a crystal lattice.

Sample	Ti-α					TiO_2_ (Anatase)					TiO				Ti_3_O_5_					
	a_0_ [Å]	c_0_ [Å]	V [%]	<D> [Å]	ε [%]	a_0_ [Å]	c_0_ [Å]	V [%]	<D> [Å]	ε [%]	a_0_ [Å]	V [%]	<D> [Å]	ε[%]	a_0_ [Å]	b_0_ [Å]	c_0_ [Å]	V [%]	<D> [Å]	ε [%]
Ref	2.9495	4.6851	100	250	−0.03	—	—	—	—	—	—	—	—	—	—	—	—	—	—	—
#1	2.9567	4.6906	8	180	−0.23	3.684	9.614	31	71	0.42	4.0686	55	600	0.49	9.7150	3.7965	9.4092	6	220	−0.10
#2	2.9473	4.6795	1	200	−0.11	3.7764	9.4892	95	91	−0.43	4.0715	3	890	0.23	9.6787	3.37989	9.4432	1	210	−0.11
#3	2.9451	4.6705	6	170	−0.23	3.7653	9.414	60	97	−0.44	4.079	30	320	0.63	9.7332	3.8042	9.4416	4	280	−0.06
#4	2.9491	4.681	12	150	−0.24	3.7804	9.5003	55	340	0.21	4.0633	33	250	0.69	—	—	—	—	—	—
#5	0.9336	4.6891	5	130	−0.33	3.7711	9.6140	72	100	−0.37	4.0794	23	140	−0.3	—	—	—	—	—	—

Another crystalline form of titanium oxide detected within the TNA structures is TiO. Phase contribution of TiO rises to 55% after first anodization, then goes down to 3% after the nanotubes are removed, and finally approaches the level between 20 and 30% in the subsequent stages of the process. Note that opposite trend can be seen considering the size of CDD particles in TiO lattice. The size equals to 600 Å in the anodized sample, increases to 890 Å within the dimples, but then falls down to 320 and even further to 140 Å at the end of the process. Except for the last stage, TiO structure is distorted by the largest tensile stresses that approach 0.23–0.69%.

Apart from the above oxides, XRD patterns also bring evidence that the films contain some small amounts of Ti_3_O_5_. Note that this polycrystalline phase occurs after first anodization but after annealing completely disappears from the samples. On the other hand, in contrast to previously mentioned oxides Ti_3_O_5_ gives a large bunch of XRD peaks, which overlap with those of pure titanium. As a result, the presence of this crystalline structure might be just an artifact of the numerical refinement method. XRD data raise question concerning current growth mechanisms and evolution of crystalline structure of TNAs. More specifically, presented results suggest that widely agreed scheme of chemical reactions leading to formation of TiO_2_ nanotubes through anodic oxidation of Ti^36^ might be incomplete as it neglects continued formation of TiO during the process. In fact, XRD measurements demonstrate substantial content of non-stoichiometric form of titanium and oxygen within aligned TNAs, especially in their external, upper parts far from the Ti substrate, whereas current models focus on kinetic equilibrium between electrochemical oxidation of Ti and dissolution of TiO_2_. It turns out, therefore, that either conversion of TiO_2_ into TiO or the field-assisted growth of TiO at the walls of nanotubes need to be considered to complete that picture. This problem needs further investigation in future works.

### PEC measurements

#### Illuminated open-circuit potential (OCP) and Flat-band potential

**Figure 5:**
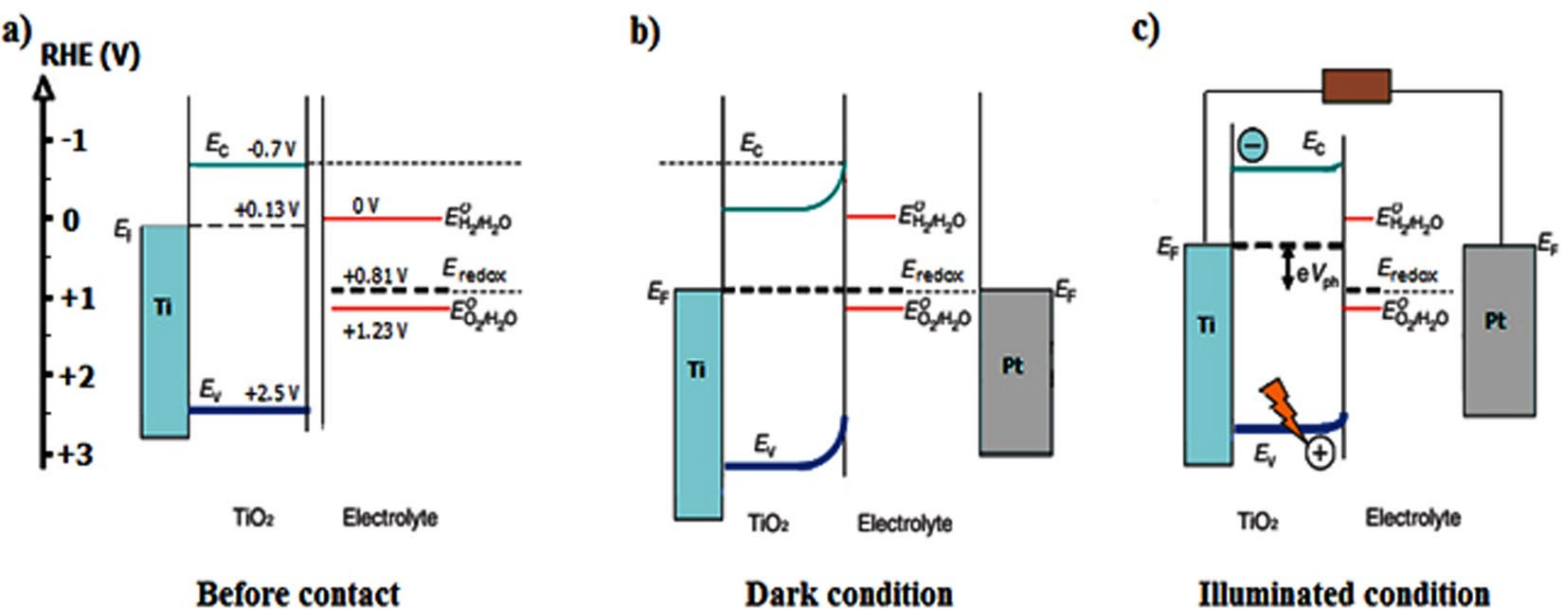
The band structure profile of a TNA electrode and the electrolyte (KOH 0.5 M) (**a**) before contact, (**b**) Equilibrium after contact in dark condition, (**c**) after over band-gap illumination in open-circuit condition. The magnitudes of potentials were achieved from OCP experiment.

**Figure 6:**
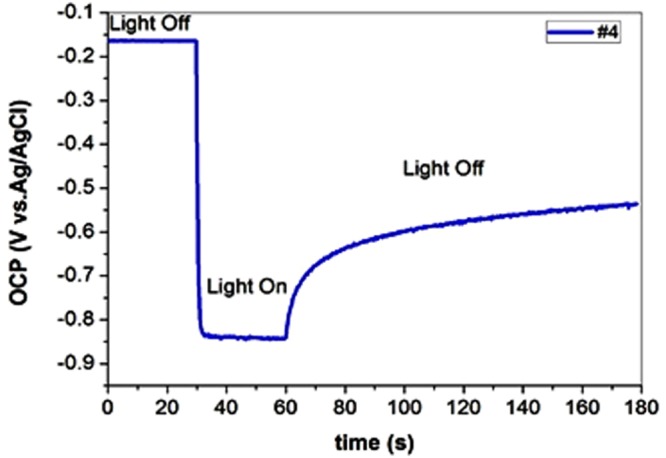
Illuminated Open-Circuit Potential (OCP) versus time under off/on cycles of light illumination of TNA electrode vs Ag/AgCl reference.

Before performing any photoelectrochemical experiment it is preferred to recognize the conductivity and the flat-band potential (*E*_fb_) of a photoelectrode. These characteristics help to reveal the band structure of a semiconductor which ultimately governs its ability to undergo effective water splitting. The illuminated open-circuit potential (OCP) is one of the most straightforward methods to estimate the *E*_fb_. Figure 5 shows schematic structure of TNA/electrolyte before and after contact (dark and illuminated) in open circuit potential conditions. The numbers on the diagram will reveal after measuring OCP in dark an illuminated condition. In the dark, when the TNA electrode and the counter-electrodes are immersed in the electrolyte, tubular domains in TNA 30–50 nm thick provide a space-charge layer inducing relevant electric field^37^. Fermi levels of both working and counter electrodes equalize with the redox potential of the electrolyte, so that the valence and conduction bands of the TNA are bent up to (*E*_f_ - *E*_redox_), where *E*_f_ is the Fermi level of the semiconductor and *E*_redox_ is the potential of the redox couples in the solution (Fig. 5b). When photoelectrode is illuminated above its band-gap (hυ > *E*_g_), photoexcited electrons and holes are generated in the conduction and the valence bands, respectively. In open circuit condition, the space-charge region in TNA creates electric field that accumulates photogenerated holes at the semiconductor/electrolyte interface and moves photoelectrons to the metal/semiconductor ohmic back contact creating non-zero photovoltage. If illumination is sufficiently intense and the carrier recombination rates in the semiconductor is not very large, the photovoltage can absolutely eliminate pre-existing band bending at the semiconductor/electrolyte interface (i.e. *E*_f_ − *E*_redox_ = *V*_ph_) (Fig. 5c). So, the upper limit of the open circuit voltage that can be achieved under irradiance represents the flat-band potential. Figure 6 shows the open-circuit voltage (*V*_oc_) of TNA (Sample #4) in dark and illuminated conditions. The negative shift in *V*_oc_ upon lighting indicates that TNA possesses n-type conductivity as predicted. Obtained flat-band potential was equal to (−0.84) V vs. Ag/AgCl reference. The difference in the *V*_oc_ of the semiconductor between dark and illumination conditions is the photovoltage *V*_ph_, which is found about 0.7 V. In other non-crystalized samples (i.e. Samples #1 and #3) non-ideal behavior was observed. Photogeneration of carriers during illumination is extremely slow and do not reach any constant value even after 350 seconds (Fig. S-1a (see supporting information)). This should be due to lack of space-charge region and proper electric field in the walls of not annealed nanotubes. Consequently, one cannot define flat-band potential in these samples. Photoexcitation of electron-hole pairs in sample #5 is also slower than in perfect TNA electrode (#4) because of high density of defect sites which play the role of recombination centers. High carrier recombination rate in this sample prevents the rapid creation of a compensating electric field and photovoltage response, and hence this sample also could not be an appropriate photoelectrode for water splitting (Fig. S-1b (see supporting information)).

In the following, the quantitative band structure of the TNA electrode (Fig. 5a) is shown. For better comparison, all potentials are converted in terms of RHE according to the Equation (5)^38^:

5 $${{\rm{E}}}_{{\rm{RHE}}}={{\rm{E}}}_{{\rm{Ag}}/{\rm{AgCl}}}+0.059{\rm{pH}}+{{{\rm{E}}}_{{\rm{Ag}}/{\rm{AgCl}}}}^{{\rm{0}}}\,({{{\rm{E}}}_{{\rm{Ag}}/{\rm{AgCl}}}}^{{\rm{0}}}=+\,{\rm{0}}{\rm{.199V}})$$

The potential level of H^+^/H_2_ is 0 V (vs RHE) and the potential level of H_2_O/O_2_ is +1.23 V. The redox potential of the electrolyte is between H^+^/H_2_ and H_2_O/O_2_ levels and closer to the H_2_O/O_2_. Figure 5 shows the *E*_f_ of semiconductor before contact with electrolyte is equal to the Flat- band potential of TNA after contact with electrolyte and under irradiation (i.e. −0.840 V vs Ag/AgCl). OCP in dark for TNA in contact with electrolyte is −0.16 V (vs Ag/AgCl) representing *E*_redox_ of electrolyte. Assuming Nernst Equation (5) in KOH electrolyte with pH = 13, *E*_redox_ = + 0.806 V (vs RHE) and *E*_fb_ = + 0.126 V for TNA. It is demonstrated that the conduction band edge of the TiO_2_ nanotubes is −0.7 V (vs RHE) in KOH with pH = 13^39^. Based on the assumption that the energy gap of TNA is 3.2 eV, the valence band edge of TNA is achieved to be +2.5 V. Figure 4a also reveals that the TNA without applying external bias is not an efficient photoelectrode for water splitting in KOH electrolyte because its Fermi level is lower than the energy level of H^+^/H_2_.

**Figure 7:**
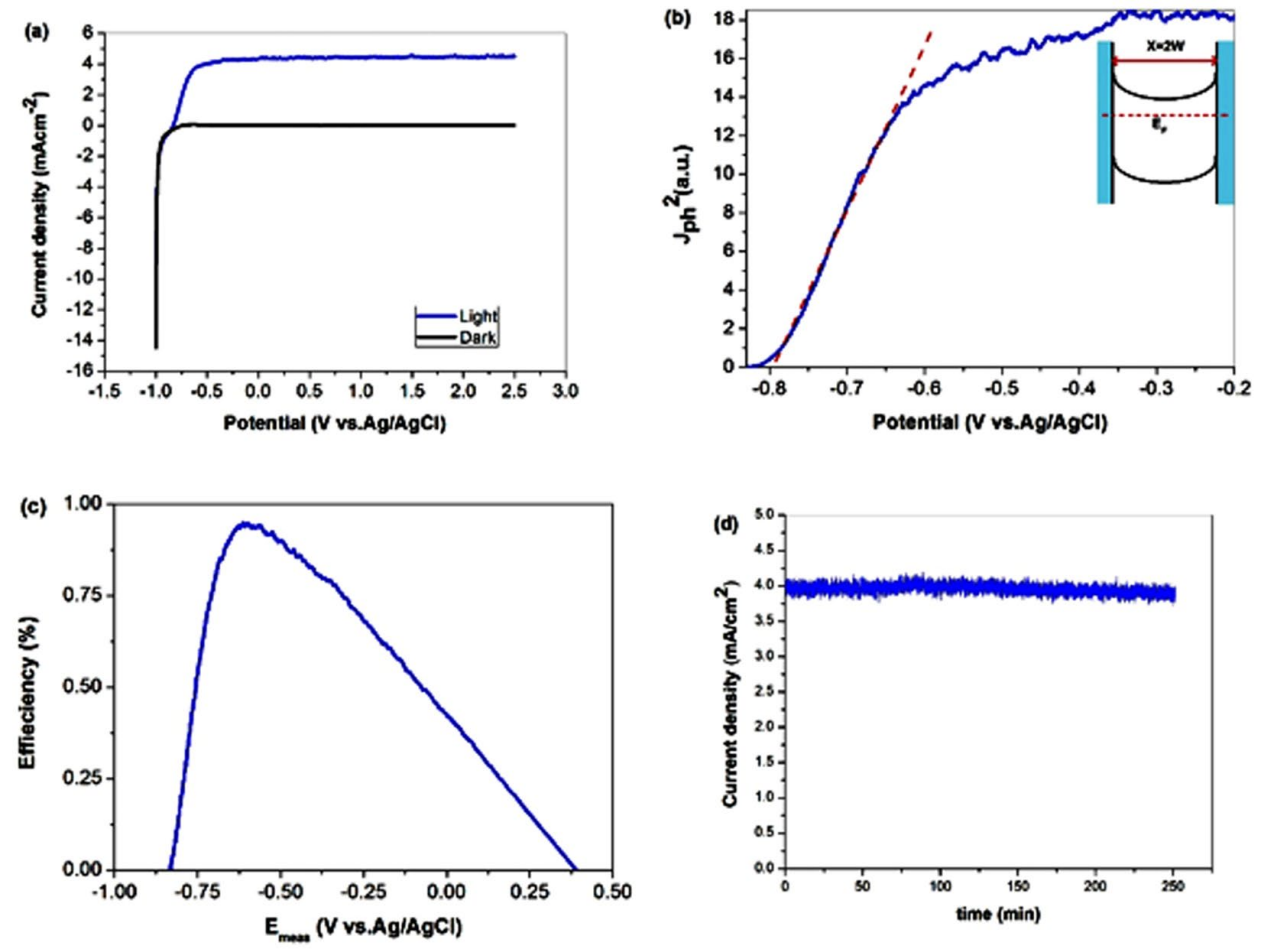
(**a**,**b**) Photocurrent density and its squared versus measured potential for TNA electrode vs Ag/AgCl reference. (**c**) Calculated photoconversion efficiency as a function of measured potential [vs Ag/AgCl]. (**d**) Photocurrent density at 0.5 V vs. Ag/AgCl potential for 250 minutes for stability measurement of TNA photoelectrode in illuminated condition.

The photocurrent density vs. voltage (J-V) characteristics induced by a Xe light source (400 mW/cm^2^) were measured with regard to Ag/AgCl reference electrode. For comparison, the dark current was also measured. For n-type semiconductor neither dark nor illuminated conditions led to generation of a current in a flat-band potential. In potentials more negative than *E*_fb_, an accumulated layer was formed and the electrode worked as cathode in both conditions. In potentials more positive than *E*_fb_ there was a depletion layer. As can be seen in Fig. 7a, there was no charge carrier in dark condition, so no oxidative current was observed. In illuminated condition, built-in electric field of the space-charge layer did not let photogenerated charges recombine at the interface and produced anodic photocurrent at external circuit. Increase in the anodic voltage changes the width of the space-charge layer according to its square root value^40^, induces more electric field, causes better e^−^/h^+^ separation and creates more photocurrent. The maximum of anodic photocurrent reaches 4 mAcm^−2^ in TNA electrode (sample #4). In the remaining samples, anodic photocurrent is negligible because of high recombination rate (Fig. S-2 (see supporting information)). This result also confirms that TNA samples without appropriate annealing (#1 and #3) and dimpled sublayer (#5) could not serve as photoelectrodes in water splitting. It is worth noting that in TNA electrode, the width of depletion layer is limited by the thickness of the walls of nanotubes (40 nm). It means that at some threshold voltage, entire wall is depleted from majority carriers (inset of Fig. 7b) and further increase in voltage has no influence on the band bending and anodic photocurrent. So, there is a plateau in the current curve. In Fig. 7b, the squared photocurrent is plotted against electrode potential. It is demonstrated that at low values of applied bias the plot follows straight line illustrative that photogenerated charges are being separated by the electric field of the space-charge layer^41–43^. As can be seen in Fig. 7b, the TNA electrode obeys this linearity over larger bias range. Indeed, substantial number of minority carriers that are generated within a distance equal to the sum of the thickness of the depletion layer and the diffusion length of the holes are able to reach the electrolyte^37^. For typical TiO_2_ nanotubes, total carrier depletion of the walls is achieved at moderate bias^44^. In our TNA electrode (Fig. 7b) the threshold voltage is about (−0.6) V vs. Ag/AgCl (~0.2 V anodic to the flat-band potential).

At higher anodic polarization, the plot deviates from linearity due to band bending^41^. The photoconversion efficiency (*η*) of light energy into chemical energy in the presence of an external potential is calculated according to^44^:

6 $$\eta ( \% )={j}_{p}\frac{{E}_{rev}^{0}-|{E}_{app}|}{{I}_{0}}\times 100$$

where ${E}_{rev}^{0}$ is the standard reversible potential (the potential corresponding to the water splitting reaction) which is 1.23 V vs. NH*E*. *E*_app_ = *E*_mea_ − *E*_fb_, the term *E*_meas_ is the electrode potential (vs. Ag/AgCl) of the working electrode at which the photocurrent was measured under illumination. *E*_fb_ is the flat-band potential (the electrode potential (vs Ag/AgCl) at open circuit conditions under the same illumination), j_p_ is the photocurrent density and I_0_ is the intensity of the incident light (400 mW/cm^2^). The results are depicted in Fig. 6c. It is apparent that the maximum photoconversion efficiency obtained for TNA electrode is about 1%. The stability of the photoelectrochemical performance of TNA electrode was measured during 250 minutes at 0.5 V vs. Ag/AgCl potential in illuminated condition. As shown in Fig. 7d, the stability test proved that the TNA electrode exhibit excellent photocurrent durability under illumination. The stability of annealed titania nanotube arrays in aqueous environment was confirmed previously^45^.

## Conclusions

Highly-ordered, smooth-mouth TiO_2_ nanotube arrays on Ti substrate were synthesized and their morphological and crystalline structures after each step of fabrication procedure were studied in details. The influence of structural modifications on the electrochemical properties of TNA layers were investigated through photoelectrochemical water splitting reaction.

According to XRD data, contribution of the base material (Ti-alpha) in the processed structures do not exceed ~10% (vol.), and they contain various forms of titanium oxides (mostly in the form of TiO and anatase TiO_2_). On the other hand, tubular forms grown during anodization procedures exhibit rather complicated microstructure: predominant TiO contribution at their upper ends and TiO_2_ (anatase) at their bottom ends. Deep analysis on surface statistical parameters shows that obtained TNAs exhibit monofractal characteristics (no long-range order spatial arrangements) except for the dimpled sublayer, at which the structures turns into bifractal ones and also, we found they are highly isotropic. The OCP measurements revealed that only annealed (450 °C) TNA layers has a potential to utilize as a photoelectrode. TNA layers without annealing with different relative phase contribution (compared to annealing samples) and grain boundaries which in turn increasing the number of charge carrier recombination centers. Because of these recombination centers photogeneration and separation of carriers during illumination is extensively slow so that space- charge region and proper electric field in the walls of non-annealed nanotubes could not form and consequently Flat- band potential and energy band structure of these samples could not be defined. In dimpled TNA layers also increasing diffracting domains creates defects in TiO_2_ structure which in turn increase the density of recombination centers. The decrement in photocurrent for the dimpled TNA layer also appears to arise from high density of defect sites which can play the role of recombination centers. OCP measurement revealed that the Flat- band potential in annealed TNA layers was obtained equal to +0.126 V vs. RHE reference. Then the band structure profile of a TNA electrode in the KOH electrolyte was measured and plotted. The photocurrent density–voltage properties induced by a Xe light source have been characterized and the maximum photoconversion efficiency about 1% was obtained for TNA electrode.

## Electronic supplementary material

Supplementary Information
