# Supplementary material for: Evolution of rough-surface geometry and crystalline structures of aligned TiO2 nanotubes for photoelectrochemical water splitting
Source: Sci Rep. 2018 Jul 18;8:10870. doi: 10.1038/s41598-018-29247-3 (PMC6052034; doi:10.1038/s41598-018-29247-3)
Supplement: Supplementary file 1 — Supplementary Information [file 41598_2018_29247_MOESM1_ESM.docx]

**Supporting Information**

**Evolution of rough-surface geometry and crystalline structures of aligned TiO2 nanotubes for photoelectrochemical water splitting**

Maryam Zare ^1^, Shahram Solaymani ^2,^ *****, Azizollah Shafiekhani^1,3^, Slawomir Kulesza ^4^, Ştefan Ţălu ^5^, Miroslaw Bramowicz ^6^

^1^ School of Physics, Institute for Research in Fundamental Sciences, PO Box 19395-5531, Tehran, Iran.

^2^ Plasma Physics Research Center, Science and Research Branch, Islamic Azad University, Tehran, Iran.

^3^ Physics Department, Alzahra University, PO Box 1993891167, Tehran, Iran.

^4^ University of Warmia and Mazury in Olsztyn, Faculty of Mathematics and Computer Science, Sloneczna 54, 10-710 Olsztyn, Poland.

^5^ Technical University of Cluj-Napoca, The Directorate of Research, Development and Innovation Management (DMCDI), Constantin Daicoviciu St., no. 15, Cluj-Napoca, 400020, Cluj county, Romania.

^6^ University of Warmia and Mazury in Olsztyn, Faculty of Technical Sciences, Oczapowskiego 11, 10-719 Olsztyn, Poland.

**Corresponding author***:

Shahram Solaymani

Plasma Physics Research Center, Science and Research Branch, Islamic Azad University, Tehran, Iran.

Phone: +989194947717

E-mail: shahram22s2000@yahoo.com

Figure S1 shows OCP measurement of titania nanotube samples before annealing ( #1 and # 3). and dimpled sublayer blue sample (#5). Figures S2 include the photocurrent measurement of titania nanotube samples before annealing ( #1 and # 3).and dimpled sublayer blue sample (#5).


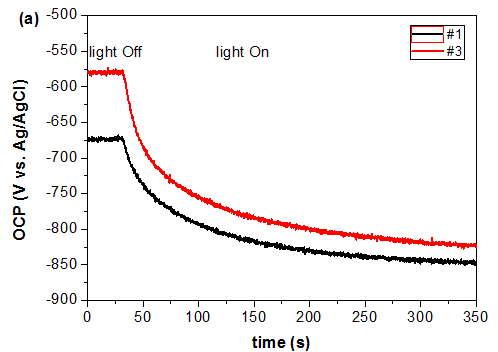


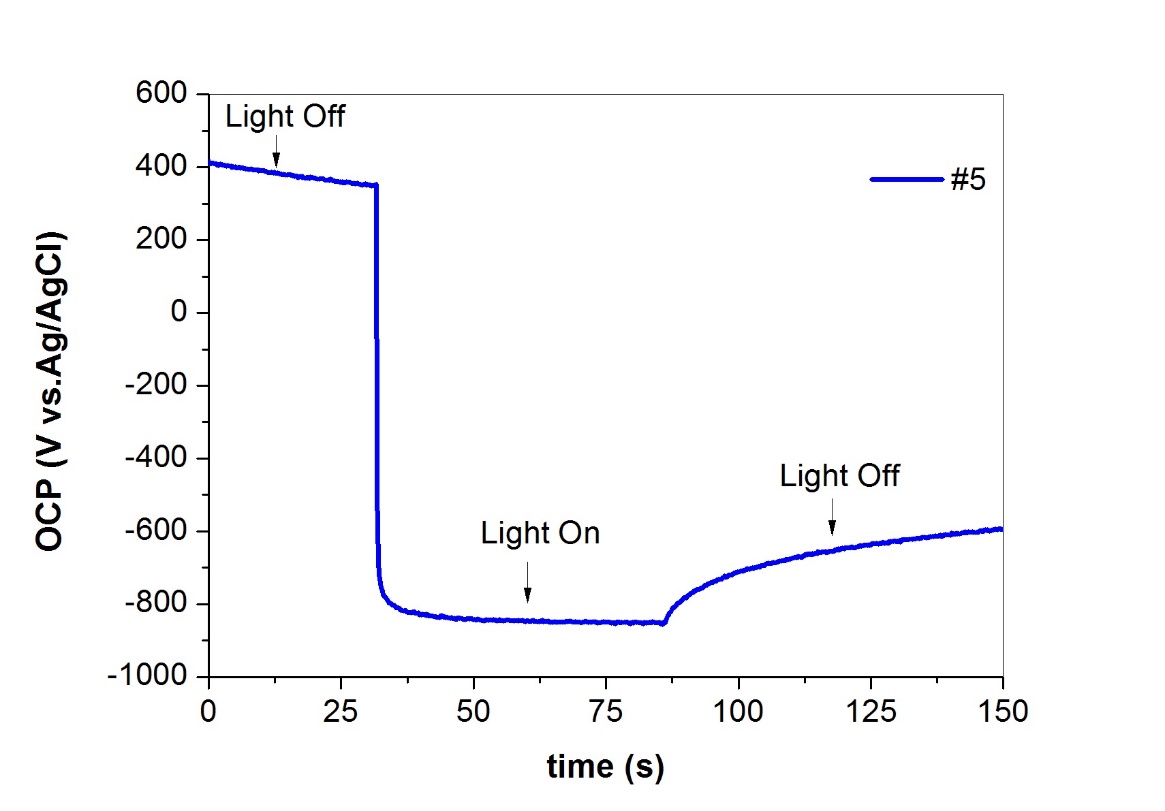


**Figure S-1**. OCP measurement of a) titania nanotube samples before annealing ( #1 and # 3). b) dimpled sublayer blue sample (#5). Photogeneration of carriers during illumination is extensively slow.


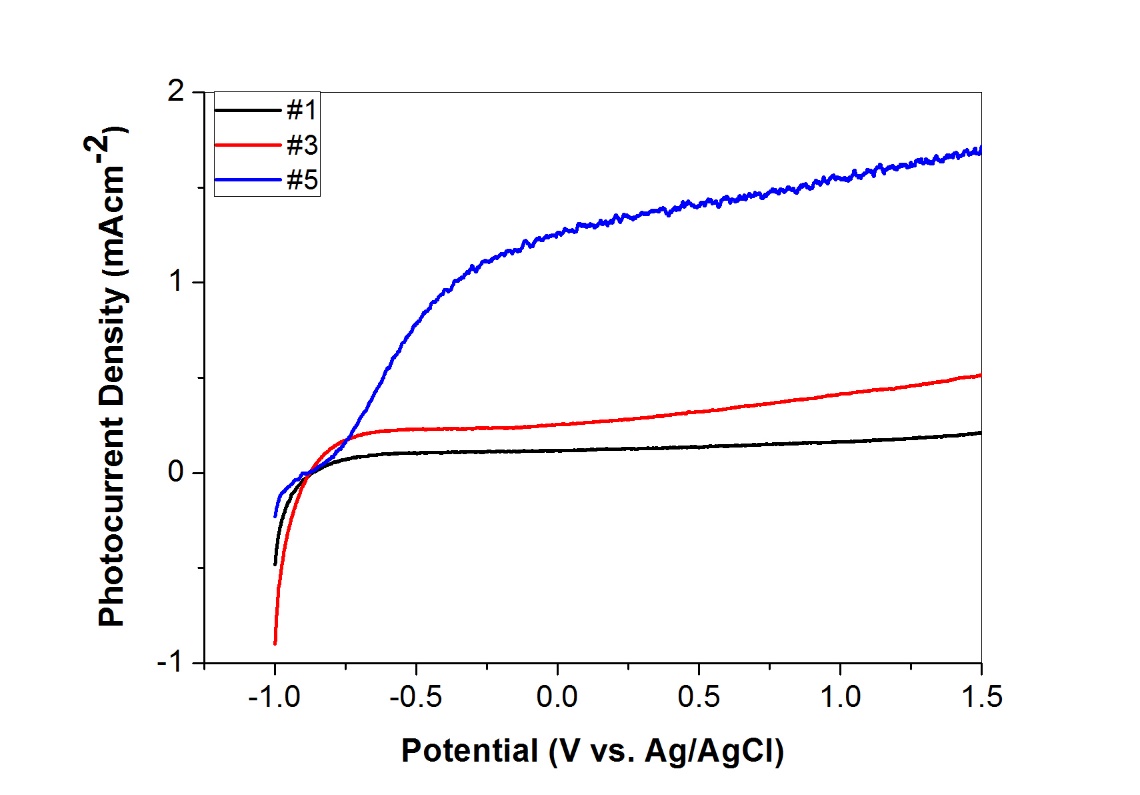


**Figure S-2**. Photocurrent measurement of titania nanotube samples before annealing ( #1 and # 3).and dimpled sublayer blue sample (#5). The anodic photocurrent is negligible because of high recombination rate.
